# A Rare Case of Cold Antibody-Mediated Autoimmune Haemolytic Anaemia in Early Systemic Lupus Erythematosus

**DOI:** 10.7759/cureus.86842

**Published:** 2025-06-27

**Authors:** Harisanth Rajaram, Sherwin Ganegoda, Rajinder Andev

**Affiliations:** 1 Internal Medicine, Royal Berkshire NHS Foundation Trust, Reading, GBR; 2 Internal Medicine, Oxford University Hospitals NHS Foundation Trust, Oxford, GBR; 3 Rheumatology, Sandwell and West Birmingham Hospitals NHS Trust, Birmingham, GBR

**Keywords:** autoimmune hemolytic anemia, cold agglutinin disease, coombs-positive anaemia, corticosteroid therapy, pediatric systemic lupus erythematosus (psle), raynaud's phenomenon, systemic lupus erythromatosus

## Abstract

Systemic lupus erythematosus (SLE) is a complex autoimmune disease characterised by autoantibody production, leading to multiorgan inflammation and tissue damage. Autoimmune haemolytic anaemia (AIHA) is a recognised haematologic complication of SLE. This report describes a 16-year-old female who presented with four weeks of generalised weakness, polyarthralgia, recurrent syncopal episodes, and Raynaud’s phenomenon. This case highlights the diagnostic challenges of SLE presenting with AIHA, underscoring the importance of prompt, comprehensive autoimmune and haematologic evaluation in adolescents with unexplained anaemia and multi-system involvement.

## Introduction

Systemic lupus erythematosus (SLE) is a chronic autoimmune disease characterised by the production of autoantibodies against various nuclear and cytoplasmic antigens, leading to widespread inflammation and tissue damage. The clinical manifestations of SLE are highly variable and can affect multiple organ systems, including the skin, joints, kidneys, heart, lungs, and central nervous system. The aetiology of SLE is multifactorial, involving genetic predispositions, hormonal influences, and environmental triggers. Viral infections have been implicated in triggering and/or exacerbating autoimmune conditions such as SLE [1]. The prevalence of SLE varies by population, with higher rates and more aggressive disease observed in women and certain ethnic groups, namely, African-Caribbean individuals [2,3]. The diagnosis of SLE is typically based on the established classification set by 2019 European Alliance of Associations for Rheumatology (EULAR)/American College of Rheumatology (ACR) criteria [4], which incorporate both clinical and immunological features such as ANA positivity, complement levels, and disease-specific antibodies. However, early presentations that lack classic clinical features can contribute to diagnostic delays and uncertainty.

Autoimmune haemolytic anaemia (AIHA) is a rare but significant haematologic complication of SLE, occurring in approximately 5-10% of patients at some point during the disease course. Cold antibody-mediated AIHA, also known as cold agglutinin disease, is an even rarer presentation of SLE, with few published case reports present in the literature [5]. 

## Case presentation

A 16-year-old female with no prior medical history presented to the Emergency Department with a four-week history of generalised weakness, fatigue, polyarthralgia, recurrent syncopal episodes, and symptoms of Raynaud’s phenomenon. Joint pain mainly involved the hands and knees, was symmetrical, and exhibited morning stiffness that improved throughout the day. Her appetite was significantly reduced, and she experienced considerable morning lightheadedness that prevented her from rising from bed. Of note, the patient’s family history was significant for diffuse cutaneous systemic sclerosis in her mother.

On initial examination, her vital signs revealed a pulse of 98 beats per minute, blood pressure of 90/50 mmHg, temperature of 36.5°C, respiratory rate of 18 breaths per minute, and oxygen saturation of 99% on room air. Physical examination demonstrated palpable cervical lymphadenopathy without hepatosplenomegaly. No dermatological signs were noted. Her menstrual history was unremarkable, and she denied any signs of gastrointestinal bleeding or haemoptysis. An urgent lymph node biopsy was performed prior to the commencement of steroids, which ruled out any infectious or malignant pathology.

Laboratory investigations revealed severe macrocytic anaemia, a positive direct Coombs test with cold agglutinins, high ANA and anti-dsDNA levels, low complement levels, and positive antiphospholipid antibodies, as summarised in Table 1, indicating SLE and a possible secondary antiphospholipid syndrome (APS). Blood film analysis revealed red cell agglutination and polychromasia. Cold antibody-mediated AIHA, a rare form of AIHA in SLE, was considered based on the presence of the above findings.

**Table 1:** Summary of relevant laboratory findings on admission MCV: mean corpuscular volume, ESR: erythrocyte sedimentation rate, CRP: C-reactive protein, LDH: lactate dehydrogenase, ANA: antinuclear antibodies

Serum	Initial Values	Reference range
Haemoglobin (g/L)	50	115-165
MCV (fL)	118.1	80-100
ESR (mm/h)	144	3-12
CRP (mg/L)	1.1	0-5
LDH (U/L)	478	125-243
Total Bilirubin (umol/L)	17	0-21
Autoimmune Profile		
Coombs test	Positive (Cold agglutinins)	Negative
ANA	>1:640	Negative
Anti-dsDNA (IU/mL)	536	0-27
Anti-Smith	Positive	Negative
C3 (mg/dL)	25	63-250
C4 (mg/dL)	<3	14-65
Beta-2 glycoprotein IgG (U/mL)	9	0-7
Beta-2 glycoprotein IgM (U/mL)	200	0-7
Anti-cardiolipin IgM (U/mL)	96	0-10
Anti-cardiolipin IgG (U/mL)	10	0-10

Inpatient treatment commenced promptly with intravenous methylprednisolone 250 mg for three days, followed by oral prednisolone 40 mg (1 mg/kg) daily, with gastrointestinal prophylaxis. During her hospital admission, the patient tested positive for COVID-19 but remained asymptomatic throughout her stay.

The patient demonstrated substantial improvement in haemoglobin levels and overall fatigue following initiation of corticosteroids, managing to avoid the need for a blood transfusion. Her rheumatology follow-up included continued hydroxychloroquine (HCQ) therapy and a titrated azathioprine regimen to aid in long-term immunomodulation. Her haemoglobin levels, joint symptoms, weakness, and lightheadedness improved with ongoing treatment.

## Discussion

The patient presented with polyarthralgia, Raynaud's phenomenon, and generalised weakness, which raised clinical suspicion of SLE, a multisystem autoimmune disease. Polyarthralgia, characterised by pain in multiple joints without significant joint swelling, is a common manifestation of SLE, affecting up to 90% of patients during the disease course. Raynaud's phenomenon, identified by colour changes in the extremities due to vasospasm, can also be associated with SLE and reflects vasculopathy or microvascular dysfunction. Generalised weakness may result from chronic inflammation, anaemia, or myopathy, which are frequently observed in SLE patients [6,7,8].

The laboratory findings included profound anaemia, elevated ANA and anti-dsDNA titres, low complement levels, and a raised ESR with a normal CRP. She also had positive anti-Smith, IgM anticardiolipin, and beta-2 glycoprotein IgM antibodies. Anaemia is a common manifestation of SLE and may result from chronic inflammation, renal involvement, or autoimmune haemolysis, as in this case [9]. Elevated ANA and anti-dsDNA titres with positive anti-smith antibodies are highly specific for SLE and indicate an autoimmune response targeting nuclear material [9]. Complement proteins (C3 and C4) are often depleted due to immune complex formation and consumption during disease activity [9]. 

The diagnosis of SLE was strongly supported by the fulfilment of the EULAR/ACR classification criteria, with this patient meeting multiple clinical and immunological domains, particularly regarding autoantibody positivity and underlying haematological involvement. 

Autoimmune diseases often share common genetic predispositions. Studies have identified several genetic loci associated with an increased risk for multiple autoimmune diseases, including systemic sclerosis (SSc) and SLE. For instance, genes such as HLA-DRB1 and other non-HLA genes like STAT4 and IRF5 are implicated in both SLE and SSc [10]. The presence of these shared genetic factors may increase the likelihood of developing an autoimmune disease if an immediate family member is affected, even if the specific diagnoses differ. This is particularly relevant in this case given the maternal history of diffuse cutaneous systemic sclerosis.

COVID-19 can exacerbate autoimmune conditions such as SLE, potentially triggering flares. The infection can induce a systemic inflammatory response, often referred to as a cytokine storm, which can activate or amplify underlying autoimmunity [11]. In patients with cold agglutinin disease (CAD), infections can exacerbate haemolytic anemia due to increased complement activation during febrile or inflammatory states. During an acute phase reaction, such as an infection, the liver produces higher amounts of complement proteins, including C4, which exacerbates the activity of the classical complement pathway and leads to increased haemolysis. In addition, microbial agents can directly activate complement, further contributing to the exacerbation of AIHA [1].

In managing SLE, particularly during relapses, the use of steroid-sparing agents like HCQ and azathioprine is crucial. HCQ is effective in reducing the frequency of SLE flares and improving long-term outcomes, while having a relatively safe profile [12]. Azathioprine, another key immunosuppressive agent, helps control disease activity and enables the tapering of corticosteroids, thereby reducing the risk of corticosteroid-related side effects such as osteoporosis, weight gain, and increased susceptibility to infection [13]. 

CAD is a form of AIHA caused by cold-reactive autoantibodies, usually IgM, which bind to erythrocytes at lower temperatures. These antibodies typically target the I antigen on erythrocytes. The binding of IgM autoantibodies activates the classical complement pathway, leading to the deposition of C3b on the red cell surface. This, in turn, facilitates the removal of affected cells by the mononuclear phagocyte system, primarily in the liver and spleen. However, the extent of haemolysis in CAD is often moderate, as IgM can detach from the red blood cells at warmer core body temperatures, preventing full complement activation and subsequent cell lysis [14,15].

In CAD, haemolysis is predominantly extravascular, meaning that the red blood cells are primarily destroyed in the spleen and liver rather than within the circulation. As a result, the release of free haemoglobin into the plasma is minimised, which leads to a reduced conversion of haemoglobin to bilirubin. Thus, bilirubin levels can remain within the normal range or be only mildly elevated [16]. 

In patients with SLE and associated AIHA, blood transfusions should be carefully considered and reserved for life-threatening situations. Repeated transfusions carry the risk of alloimmunisation, where the patient develops antibodies against transfused blood cells, complicating future transfusion therapy due to the increased likelihood of haemolysis [17].

The presence of antiphospholipid antibodies should be re-evaluated after 12 weeks to confirm the diagnosis of APS. This is essential, as a single positive test can occur transiently due to infections or other nonspecific factors. Persistently positive antiphospholipid antibodies, combined with relevant clinical criteria, confirms APS, which requires specific management to prevent thrombotic events [18].

## Conclusions

This case illustrates a rare presentation of SLE with cold antibody-mediated AIHA. Early recognition and comprehensive autoimmune work-up were critical in diagnosing SLE in the presence of severe anaemia and multi-system involvement. Prompt initiation of immunosuppressive therapy, including corticosteroids and hydroxychloroquine, led to clinical improvement. This case highlights the need for vigilance in evaluating young patients with unexplained haemolytic anaemia, as SLE with atypical presentations can pose diagnostic challenges but respond well to timely intervention.
